# The Causal Effects of Lipid Profiles on Sleep Apnea

**DOI:** 10.3389/fnut.2022.910690

**Published:** 2022-06-21

**Authors:** Hongyi Tang, Qing Zhou, Fu Zheng, Tong Wu, Yi-Da Tang, Jiuhui Jiang

**Affiliations:** ^1^Department of Orthodontics, Peking University School and Hospital of Stomatology, Beijing, China; ^2^Department of Cardiology, State Key Laboratory of Cardiovascular Disease, National Center for Cardiovascular Diseases, Fuwai Hospital, Chinese Academy of Medical Sciences and Peking Union Medical College, Beijing, China; ^3^Department of Cardiology, Graduate School of Peking Union Medical College, Chinese Academy of Medical Sciences and Peking Union Medical College, Beijing, China; ^4^Department of Cardiology and Institute of Vascular Medicine, Peking University Third Hospital, Beijing, China; ^5^Key Laboratory of Molecular Cardiovascular Science, Ministry of Education, Beijing, China

**Keywords:** genetics, sleep apnea, lipid profiles, multivariable Mendelian randomization, triglyceride

## Abstract

**Introduction:**

Observational studies have suggested that lipid profiles were associated with risk of sleep apnea (SA). However, the specific lipid types and whether this relationship has a causal effect are uncertain. This study conducted two-sample Mendelian randomization (MR) and multivariable Mendelian randomization (MVMR) to investigate the potential causal relationship between lipid profiles and risk of SA.

**Materials and Methods:**

We used the largest genome-wide association study (GWAS) on European participants on the UK Biobank. After a rigorous single nucleotide polymorphism screening process to remove confounding effects, we performed MR and MVMR to explore the causal relationship between lipid profiles and SA risk.

**Results:**

Both MR and MVMR showed causal effects of increased triglyceride on SA risk [MR: per 10 units, odds ratio (OR): 1.0156; 95% CI: 1.0057–1.0257; *P* value = 0.002; MVMR: per 10 units, OR: 1.0229; 95% CI: 1.0051–1.0411; *P* value = 0.011]. The sensitivity analysis including Cochran’s Q test, MR-Egger intercept, and MR pleiotropy residual sum and outlier (MR-PRESSO) test indicated that our findings were robust. The causal effects of triglyceride on SA did not change after adjusting for potential confounders (obesity, age, sex, and airway obstruction).

**Conclusion:**

Genetically increased triglyceride levels have independent causal effects on risk of sleep apnea without the confounding effects of obesity, suggesting that lowering triglyceride concentrations may help to reduce the risk of sleep apnea.

## Introduction

Sleep apnea (SA) is defined as the absence of inspiratory airflow for at least 10 s during sleep (1) and is associated with cardiovascular disease (2), stroke (3), diabetes (4), increased traffic accidents (5), lost workdays (6), and even death (7). Thus, prevention of SA is necessary, as it might cause huge social and personal burden.

Lipid profiles are an important component of human metabolism. Recent investigations have focused on the potential link between lipid profiles and SA (8–11). Obstructive sleep apnea (OSA) is the most prevalent type of SA (2). A meta-regression analysis reported that patients with OSA appeared to have elevated levels of total cholesterol, triglyceride and low-density lipoprotein (LDL), and decreased level of high-density lipoprotein (HDL) (8). A twin study demonstrated that the co-occurrence of OSA and hypertriglyceridemia had a genetic influence (9). Another cross-sectional study reported that the association between dyslipidemia and SA was limited to severe patients (10). Despite previous studies suggesting there was an association between lipid profiles and SA, the results were mainly based on observational studies and meta-analyses, which were prone to systematic bias and could not explore the causal relationship.

The causal relationship between lipid profiles and SA needs to be further explored. There are challenges in carrying out this exploration. The causal relationship between lipid profiles and SA might be confounded by other factors. Obesity is associated with lipid profiles (12) and is also considered to be a major factor contributing to SA (11, 13). This makes it difficult to explain whether the association is a causal relationship between lipid profiles and SA or the relationship comes from their common link with obesity. Additionally, common lipid profile test items include triglyceride, LDL, HDL, apolipoprotein A-1 (ApoA-1), and apolipoprotein B (ApoB). Their mutual influences may also interfere with the exploration of specific components significantly related to SA and lipid profiles. Mendelian randomization (MR) has become a reliable method to estimate for causal relationship (14) and is less prone to conventional confounding issues because of random assortment of alleles (15). Recently, multivariable Mendelian randomization (MVMR) has emerged as a method that allows for simultaneous assessment of separate but correlated exposures (16).

In this study, we extracted genetic instruments using summary statistics from a large-scale genome-wide association study (GWAS) on lipid profiles (triglyceride, LDL, HDL, ApoA-1, and ApoB) and SA. We conducted two-sample MR to investigate the causal relationship between lipid profiles and risk of SA. We carried out MVMR to explore more strong evidence for an independent causal effect of each component of lipid profiles on SA.

## Materials and Methods

### Selection of Data Sources

In this study, we performed two-sample MR as well as MVMR analysis of lipid profiles (triglyceride, LDL, HDL, ApoA-1, and ApoB) and SA using summary statistics from a large-scale genome-wide association study (GWAS). All original GWASs received ethical permission from corresponding ethics committees, and related participants signed informed consent (17). However, ethics permission was not required for the present study because it was derived from summary statistical data.

Genetic datasets of lipid profiles (triglyceride, HDL, LDL, ApoA-1, and ApoB) were retrieved from the largest GWAS (sample size: triglyceride: *N* = 441,016, HDL: *N* = 403,943, LDL: *N* = 440,546, ApoA-1: *N* = 393,193, and ApoB: *N* = 439,214), whose participants were of white European ancestry, on the UK Biobank (UKB) (17, 18). We selected all single nucleotide polymorphisms (SNPs) that reached genome-wide significance (*P* < 5 × 10^–8^), removed relative SNPs with stringent linkage disequilibrium (LD) (19) (default of the LD_clump function: R^2^ = 0.001, clumping window = 10,000), and obtaining the primary instrumental variables: triglyceride, HDL, LDL, ApoA-1, and ApoB. The primary instrumental variables, triglyceride, HDL, LDL, ApoA-1, and ApoB, consisted of 313, 362, 177, 299, and 198 SNPs, respectively. Because a large number of studies have shown that obesity is one of the main factors causing SA (11, 13, 20, 21) and that the occurrence of obesity is closely related to lipid metabolism (12, 22), we pruned all SNPs related to obesity from the primary instrumental variables to remove the confounding effects of obesity. We searched the traits of all the preliminary screened SNPs from PhenoScanner, which is a database of human genotype-phenotype associations.^1^ The following traits were selected as potential confounders because they are associated with obesity: body mass index, whole body fat mass, weight, and body fat percentage. SNPs that were not found on PhenoScanner were also removed (no search results). After the removal of SNPs associated with the potential confounders, 255, 305, 158, 237, and 169 SNPs remained in the instrumental variables triglyceride, HDL, LDL, ApoA-1, and ApoB, respectively. The screened SNPs associated with the potential confounders can be viewed online in Supplementary Table 1.

We used the data of SA from the MR-data database (23).^2^ We searched using “sleep apnea” as the keyword, selecting data that used participants of European descents in the database. We chose the largest sample size datasets of SA in the MRC Integrative Epidemiology Unit (MRC-IEU) consortium^3^ from the UKB (18) (dataset ID: ukb-b-7853, *N* = 463,010, diagnosis: sleep apnea). Apnea-hypopnea index (AHI) is defined as the frequency of obstructive or mixed apnea or hypopnea per hour and is the disease-defining threshold for laboratory diagnosis of sleep apnea obtained by laboratory polysomnography test (24, 25). The diagnosis of SA is determined by AHI > 5/h for adults (26). Pediatric SA is diagnosed as AHI ≥ 1/h or obstructive hypoventilation, manifested by Pa CO_2_ > 50 mm Hg for 25% of sleep time, coupled with snoring, paradoxical thoracoabdominal movement, or flattening of nasal airway pressure waveform (27).

**FIGURE 1:**
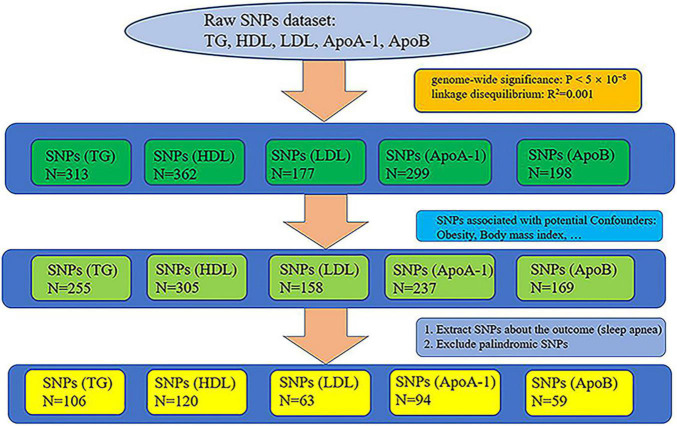
Whole process of extracting single nucleotide polymorphisms (SNPs).

We extracted the SNPs of the instrumental variables of lipid profiles (triglyceride, HDL, LDL, ApoA-1, and ApoB) from the dataset of sleep apnea and excluded palindromic SNPs, acquiring 106, 120, 63, 94, and 59 SNPs, respectively. The whole process of extracting SNPs is shown in Figure 1. The final extracted SNPs can be viewed online in Supplementary Table 2.

### Mendelian Randomization Analysis

**FIGURE 2:**
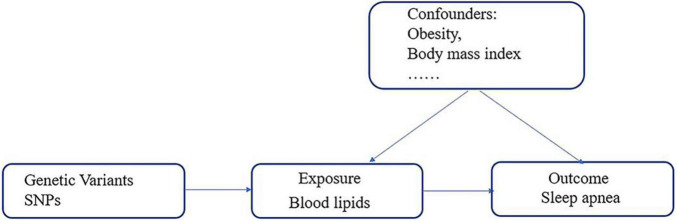
Schematic representation of two-sample Mendelian randomization.

We conducted five independent two-sample MR analyses to evaluate the potential causal relationship between lipid profiles (triglyceride, HDL, LDL, ApoA-1, and ApoB) and risk of SA. MR analysis is based on the following three assumptions (28, 29): (1) the genetic variables must be closely related to the exposure, (2) the variables must affect the outcome only through their effects on the exposure, and (3) the variables must be independent of any confounding factors of the association between the exposure and the outcome (Figure 2).

We performed an inverse variance-weighted (IVW) meta-analysis of each Wald ratio for each SNP (15). Applying fixed effects IVW requires the assumption that there is no heterogeneity or horizontal pleiotropy in the SNPs (15). We also used maximum likelihood method to estimate the causal effect. The method maximized the likelihood given the SNP-exposure and the SNP-outcome effects directly and assumed a linear regression between the exposure and the outcome (15). Leave-one-out, scatter, forest, and funnel plots were produced to illustrate the results.

**FIGURE 3:**
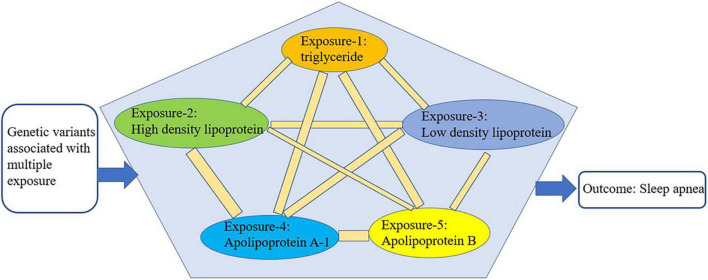
Schematic representation of multivariable Mendelian randomization.

We conducted multivariate Mendelian randomization (MVMR) to eliminate the interaction among the selected exposures (triglyceride, HDL, LDL, ApoA-1, and ApoB) that could be confounding factors (16) (Figure 3). For the MVMR analysis, we excluded any SNPs with LD (R^2^ > 0.001, clumping window = 10,000) and SNPs being palindromic with intermediate allele frequencies, retaining 433 SNPs. In addition, it should be considered that SA is also closely related to age, gender, and airway obstruction. Therefore, after finding the specific lipid type with significant causal effects on SA from the lipid profiles, we conducted a multivariable Mendelian randomization analysis to analyze its effects on SA adjusted for age, gender, and airway obstruction. As no available GWAS on age could be found, we used telomere length as substitute for age, because a wide range of studies have manifested that telomere length is largest at birth and decreases with age, and that it is a biomarker of aging (30). Therefore, the traits of GWAS representing age, gender, and airway were telomere length, genetic sex, and chronic obstructive airway disease, respectively. The GWASs mentioned above were from the MR-data database (see text footnote 2) (23). The dataset IDs of the three GWASs are as follows: ieu-b-4879 (telomere length), ukb-d-is_female (sex), and ukb-b-13447 (obstructive airway disease). All the analyses were performed using R version 4.0.5 (RStudio).

### Sensitivity Analysis

To further assess the robustness of the findings, we performed a Cochran’s Q test to assess the heterogeneity between the genetic variables (31). Horizontal pleiotropy occurs when genetic instruments are associated with more than one independent biological pathway, which can lead to a violation of the fundamental assumption of the MR study (i.e., the variable is not related to the outcome other than *via* the confounding factors) (32). We conducted an MR–Egger intercept test to evaluate the horizontal pleiotropy (33). An MR pleiotropy residual sum and outlier (MR-PRESSO) test was performed to provide outlier removal, verify the results, and assess the horizontal pleiotropy (34). We applied F statistic to evaluate instrument strength (35). F statistic was calculated with the formula (35) F = (N − k − 1)/N × R^2^/(1 − R^2^) (N = sample size, k = the number of selected SNPs, R^2^ represents the phenotype variance induced by the SNPs.) When R^2^ is not available, we used the formula R^2^ = 2 × MAF × (1 − MAF) × (beta/SD)^2^ (beta = the effect value of the genetic variant in the exposure (35), MAF = the effect allele frequency (30), SD (standard deviation) = SE × √N, SE = the standard error of the genetic variant in the exposure, N = sample size).

## Results

### Mendelian Randomization Estimates

**FIGURE 4:**
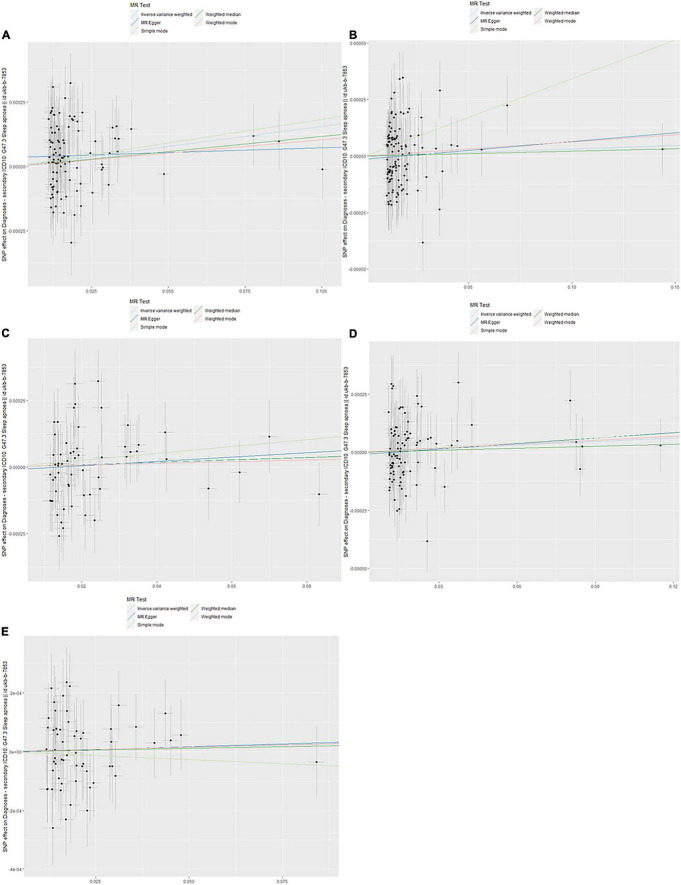
Scanner plot. **(A)** Triglyceride, **(B)** high-density lipoprotein, **(C)** low-density lipoprotein, **(D)** apolipoprotein A-1, and **(E)** apolipoprotein B.

**FIGURE 5:**
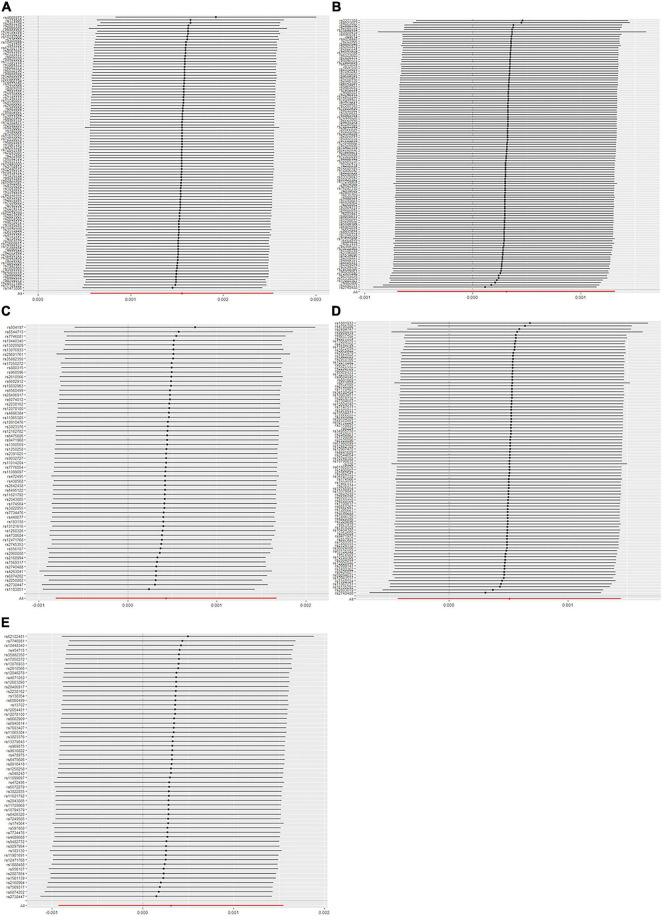
Leave-one-out plot. **(A)** Triglyceride, **(B)** high-density lipoprotein, **(C)** low-density lipoprotein, **(D)** apolipoprotein A-1, and **(E)** apolipoprotein B.

The evaluation for the association between lipid profiles and SA risk is shown in Table 1. The two-sample MR analysis found that increased triglyceride levels showed a causal association with risk of SA based on inverse-variance weighting. The OR (odds ratio) indicated that a 10-unit increase in triglyceride was causally associated with a 1.56% increase in SA risk (Table 1, triglyceride: *N* = 106 SNPs, OR: 1.0156, 95% CI: 1.0057–1.0257, *P* = 0.002). No causal relationship was found between the other lipids (HDL, LDL, ApoA-1, and ApoB) and SA risk (Table 1, HDL: *N* = 120 SNPs, OR: 1.0031, 95% CI: 0.9931–1.0132, *P* = 0.544; LDL: *N* = 63 SNPs, OR: 1.0043, 95% CI: 0.992–1.0168, *P* = 0.494; ApoA-1: *N* = 94 SNPs, OR: 1.0051, 95% CI: 0.9957–1.0146, *P* = 0.289; ApoB: *N* = 59 SNPs, OR: 1.0031, 95% CI: 0.9908–1.0156, *P* = 0.623). The maximum likelihood method showed similar results (Table 1, triglyceride: OR = 1.0157, 95% CI: 1.0059–1.0256, *P* = 0.001; HDL: OR = 1.0031, 95% CI: 0.9939–1.0124, *P* = 0.508; LDL: OR = 1.0044, 95% CI: 0.9929–1.016, *P* = 0.456; ApoA-1: OR = 1.0051, 95% CI: 0.9961–1.0141, *P* = 0.267; ApoB: OR = 1.0031, 95% CI: 0.9907–1.0156, *P* = 0.623). In the MVMR analysis, increased triglyceride still showed a causal effect on SA risk and the OR of the MVMR indicated that a 10-unit increase in triglyceride was causally associated with a 2.29% increase in SA risk (Table 2, triglyceride, OR: 1.0229, 95% CI: 1.0051–1.0411, *P* = 0.011; HDL, OR: 1.0128, 95% CI: 0.9708–1.0565, *P* = 0.556; LDL, OR: 1.004, 95% CI: 0.9463–1.0651, *P* = 0.895; ApoA-1, OR: 0.9902, 95% CI: 0.9498–1.0323, *P* = 0.642; ApoB, OR: 0.9866, 95% CI: 0.932–1.0444, *P* = 0.641). In the MVMR analysis related to age, sex, and airway obstruction, the causal effects of triglyceride on SA adjusted for age, gender, and upper airway obstruction were still significant (triglyceride adjusted for telomere length, OR: 1.0142, 95% CI: 1.005–1.0234; triglyceride adjusted for genetic sex, OR: 1.0169, 95% CI: 1.0082–1.0256; triglyceride adjusted for obstructive airway disease, OR: 1.0171, 95% CI: 1.0086–1.0256). The scatter plots illustrated the causal relationship between triglyceride and SA risk (Figure 4). The leave-one-out plots of triglyceride illustrated that even if any SNP in the instrumental variables was removed, the rest of the data could still achieve a significant causal effect (Figure 5). The forest plots showed causal effects of triglycerides on risk of SA, and all the funnel plots showed no asymmetry (Supplementary Figures 1, 2). In addition, the F statistics of all the instrumental variables were greater than 10.

**TABLE 1:** Mendelian randomization estimates lipid profiles and sleep apnea.

Inverse-variance weighted method						
Traits (per 10 units)	*N*	F	R^2^	OR	95% CI	*P*-Value
TG	106	31.7032	0.0157	1.0156	1.0057–1.0257	0.002
HDL	120	24.1751	0.0186	1.0031	0.9931–1.0132	0.544
LDL	63	26.6363	0.0094	1.0043	0.9920–1.0168	0.494
ApoA-1	94	31.1115	0.0187	1.0051	0.9957–1.0146	0.289
ApoB	59	21.1138	0.0082	1.0031	0.9908–1.0156	0.623

**Maximum likelihood method**						

**Traits**						
TG				1.0157	1.0059–1.0256	0.001
HDL				1.0031	0.9939–1.0124	0.508
LDL				1.0044	0.9929–1.0160	0.456
ApoA-1				1.0051	0.9961–1.0141	0.267
ApoB				1.0031	0.9907–1.0156	0.623

**TABLE 2:** Multivariable Mendelian randomization estimates lipid profiles and sleep apnea.

Traits (per 10 units)	*N* = 433	OR	95% CI	*P*-Value
TG		1.0229	1.0051–1.0411	0.011
HDL		1.0128	0.9708–1.0565	0.556
LDL		1.0040	0.9463–1.0651	0.895
ApoA-1		0.9902	0.9498–1.0323	0.642
ApoB		0.9866	0.9320–1.0444	0.641

### Sensitivity Analysis

The Q variable showed no heterogeneity for the analysis of triglyceride, HDL, LDL, ApoA-1, and ApoB and SA (Table 3, MR-Egger Q, triglyceride: *P* = 0.427, HDL: *P* = 0.068, LDL: *P* = 0.159, ApoA-1: *P* = 0.224, and ApoB: *P* = 0.659). The MR-Egger intercept did not find significant horizontal pleiotropy for all the analyses mentioned above (Table 3, Egger intercept, triglyceride: *P* = 0.086, HDL: *P* = 0.458, LDL: *P* = 0.705, ApoA-1: *P* = 0.611, and ApoB: *P* = 0.949). The MR-PRESSO showed no outliers in all the instrumental variables. The estimates of the MR-PRESSO found causal effects of increased triglycerides on SA risk, in agreement with the results of the IVW and maximum likelihood methods (Table 4, triglyceride: *P* = 0.019, HDL: *P* = 0.494, LDL: *P* = 0.702, ApoA-1: *P* = 0.292, and ApoB: *P* = 0.597).

**TABLE 3:** The estimates of Q test and MR-Egger intercept.

Traits	Q test (Q *P*-Value)	MR-Egger intercept (*P*-Value)
TG	0.427	0.086
HDL	0.068	0.458
LDL	0.159	0.705
ApoA-1	0.224	0.611
ApoB	0.659	0.949

**TABLE 4:** Mendelian randomization pleiotropy residual sum and outlier test (MR-PRESSO) estimates between lipid profiles and SA.

		Raw estimates			Outlier-corrected estimates		
Traits (per 10 units)	*N*	OR	95% CI	*P*-Value	OR	95% CI	*P*-Value
TG	106	1.0118	1.0020–1.0216	0.019	NA	NA	NA
HDL	120	1.0035	0.9936–1.0135	0.494	NA	NA	NA
LDL	63	1.0023	0.9904–1.0144	0.702	NA	NA	NA
ApoA-1	94	1.0050	0.9957–1.0143	0.292	NA	NA	NA
ApoB	59	1.0031	0.9916–1.0148	0.597	NA	NA	NA

## Discussion

In this study, we applied both two-sample Mendelian randomization (MR) and multivariable Mendelian randomization (MVMR) to demonstrate a causal relationship between genetically increased triglyceride and risk of sleep apnea (SA). The estimates of heterogeneity and horizontal pleiotropy showed that our results were robust. The finding of the MR-PRESSO was in agreement with the inverse variance weighting (IVW) and maximum likelihood, further strengthening the robustness of our results. The MR-PRESSO estimates found no outliers, which indicated the stability of our instrumental variables. The F statistics of all the instrumental variables were greater than 10, showing a strong association between the SNPs and the exposure with sufficient statistical power (35).

Many previous studies have demonstrated the association between risk of SA and lipid profiles including LDL, HDL and triglyceride (8–10, 36, 37). Continuing from previous relative studies, our findings showed causal effects of triglycerides on risk of SA on the basis of MR analysis. To our knowledge, this study is the first to investigate the relationship between lipid profiles and SA risk from the perspective of causality.

Our study revealed that the specific lipid causing SA was triglyceride, which is consistent with a variety of previous studies. For example, a study evaluating the association between triglyceride-glucose index (TyG index) and OSA found that higherTyG index was independently associated with increased OSA risk (OR = 3.348, 95% CI = 1.081–10.372, *P* < 0.05) (38). TyG was calculated using the following equation: ln [fasting triglyceride (mg/dl) × fasting glucose (mg/dl)/2]. The study also suggested a predictive role of TyG in OSA onset. Another study evaluated the visceral adipose tissue (VAT) and subcutaneous adipose tissue (SAT) of 41 participants with OSA (22 men and 19 women) and 39 controls (20 men and 19 women). In the men, apnea was associated with VAT, whereas in the women it was associated with subcutaneous, visceral, and total fats (39). These findings suggested the need for sex-specific therapeutic strategies such as reduction of visceral fat through exercise or pharmacological treatment in men and weight loss in women. In another study, Lebkuchen et al. used metabolomic and lipidomic strategies to select potential biomarkers for OSA. From 22 lipids initially selected, glycerophosphoethanolamines, sphingomyelin, and lyso-phosphocholines proved to be best associated with OSA and were considered to be potential early biomarkers in OSA screening (40). In terms of mechanisms linking lipids with OSA, a study assessed the AHI, inflammatory factors, and precise body fat of 392 adolescents to explore the mediation effects of systemic inflammation in the linkage between visceral adiposity and incident OSA. The study found that 42% of the association between visceral fat and OSA in the adolescents was mediated by interleukin-6 (IL-6) (*P* = 0.03), and that 82% of the association was mediated by C-reactive protein (CRP) (*P* = 0.01), suggesting that inflammation is a mediator in the causal relationship between body fat and SA (41). The studies mentioned above conducted various laboratory tests and manifested the link between lipids and SA, which makes our conclusion more convincing and robust, and pointed out directions for future research.

**FIGURE 6:**
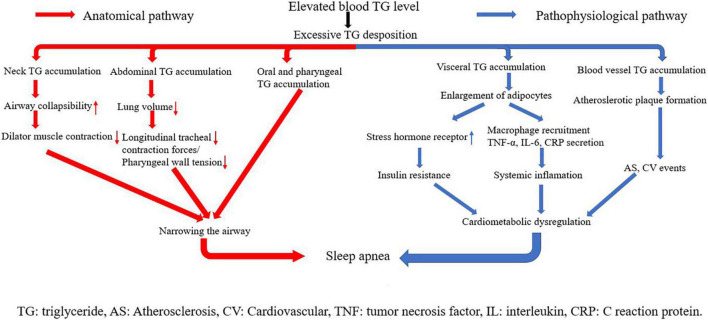
Underlying mechanisms linking triglyceride with sleep apnea.

The underlying mechanisms linking triglyceride and SA can be classified into two parts: anatomical pathways and pathophysiological pathways (Figure 6). Triglyceride is any of a class of compounds that consist of an ester of glycerol with three fatty acids. Most natural fats and oils are triglycerides. When blood triglyceride level is elevated, there is an excessive triglyceride deposit on various parts of the human body. The accumulation of triglyceride from blood is the key point in the linkage between blood triglyceride level and SA. Three kinds of triglyceride accumulation constitute the anatomical pathways. (1) Oral and pharyngeal triglyceride accumulations narrow the airway directly (42–45). The fat tissue under the mandible, in the tongue, soft palate, and uvula largely compresses the anatomical space for respiration in the oral cavity and pharynx. Patients are able to compensate for the upper airway narrowing by increasing the activity of upper airway muscles during wakefulness. However, this protective effect is lost during sleep because of relaxation of muscles (42). (2) Abdominal fat accumulation including subcutaneous fat and visceral fat that surround visceral organs in the abdominal cavity markedly increases abdominal pressure, leading to reduction in lung volume. Reduction of lung volume may decrease longitudinal tracheal traction forces and pharyngeal wall tension, which is predisposed to narrowing of the airway (46). (3) Neck fat accumulation increases the collapsibility of the upper airway, decreases the efficiency of dilator muscle contraction (47), and leads to sarcopenia, denervation, and skeletal muscle dysfunction. These changes are also linked with upper airway narrowing and SA (48).

Visceral and vascular triglyceride accumulations are crux of pathophysiological pathways. Higher levels of visceral fat have been observed in both obese and non-obese men with OSA, even when matched with controls for age and BMI (39, 49). When blood triglyceride level is elevated, adipocytes in visceral fat tissues grow quite large before dividing (50), and the size of lipid droplets in adipocytes increases a lot. The change makes visceral adipose tissues have more glucocorticoid and adrenergic and androgen receptors, and become more susceptible to glucocorticoid, catecholamine, and androgen signaling (51). In addition, adipocytes with abnormal large lipid droplets uptake glucose at a high rate and develop to insulin resistance (52). Insulin resistance represents an important mechanism of cardiometabolic dysfunction, and SA has long been considered as a common epiphenomenon of cardiometabolic dysfunction. In addition, along with weight gain, adipocytes themselves begin secreting tumor necrosis factor α (TNF-α). TNF-α stimulates surrounding endothelial cells to produce monocyte chemoattract protein-1 (MCP-1), which promotes macrophage recruitment. Macrophages secret many kinds of cytokines including IL-6 and IL-1. As a result, plasma CRP and other kinds of acute phase reactive proteins are elevated, triggering systemic inflammation (53, 54). Inflammatory response plays a causal role in metabolic dysregulation and subsequent development of SA. Excessive blood triglyceride can also deposit in blood vessels directly, especially where endothelia are destroyed. This kind of deposition is a main trigger of atherosclerotic plaque formation, leading to atherosclerosis and arterial stiffness. Atherosclerosis exacerbates organ ischemia and hypoxia, which is one of the main causes of various cardiovascular diseases. Again, cardiometabolic dysregulation is triggered in this pathway, and then SA happens (55, 56).

We have clarified how triglycerides play a causal role in the pathogenesis of SA and why LDL, HDL, ApoA-1, and ApoB have no causal effects on SA needs to be explained. The reasons can be listed as follows: 1. Triglycerides are hydrophobic, non-polar neutral molecules. This kind of structure makes triglycerides water-insoluble and easy to get together and deposit in extracellular fluid. However, HDL, LDL, ApoA-1, and ApoB have hydrophilic polar groups. HDL and LDL have a central hydrophobic core of non-polar lipids, primarily cholesterol esters and triglycerides. The hydrophobic core is surrounded by a hydrophilic membrane consisting of phospholipids, free cholesterol, and apolipoproteins directed outward (57). ApoA-1 and ApoB also have hydrophilic amino acid sequences (58). The hydrophilic parts make them soluble in the salt-water-based internal environment. Therefore, even if the levels are elevated, it is hard for them to deposit and accumulate in the oral cavity, pharynx, neck, and abdomen to narrow the airway, and neither can they form fat tissues to cause metabolic dysregulation like triglycerides. In brief, their hydrophilic structures suggest that they are not able to have an obvious direct causal effect on SA like triglycerides. 2. The function of LDL and HDL is transporting cholesterol instead of triglycerides. ApoA-1 and ApoB are major apolipoproteins of HDL and LDL, respectively (59). Fluctuation in levels of LDL and HDL has a direct influence on the level of cholesterol but hardly affects triglycerides. In terms of lipid component, triglycerides only account for 12% of total lipid content of both LDL and HDL, while cholesterol esters account for 59 and 40% of total lipid content of LDL and HDL, respectively (60). Cholesterol, the major core lipid of LDL and HDL, is an essential structural component of the cell membrane, where it is required to establish proper membrane permeability and fluidity (61). In addition, cholesterol is an important component for the manufacture of steroid hormones, bile acids, and vitamin D (62). Cholesterol also has hydrophilic group (-OH) and is not as easy as to accumulate as triglycerides. Therefore, the physiological function of HDL, LDL, ApoA-1, and ApoB suggests that they are unlikely to cause SA indirectly through triglyceride pathways.

From the perspective of diet, the content of triglycerides in the body is directly related to the intake of exogenous glucose and fats, which suggests that reducing the risk of SA can be achieved by decreasing the intake of exogenous triglycerides. Relative studies reported that the optimal diet consisting of not more than 50–60% carbohydrate sources, comprising mostly complex carbohydrates such as whole grains and fruits and vegetables, was an effective management to lower triglyceride concentrations (63). Besides optimal diet, regular aerobic exercise, avoiding alcohol abuse, and drug treatment including fibrates, omega-3 fatty acids, niacin, and statins were also important factors in the prevention and treatment of hypertriglyceridemia (63).

It should be noted that obesity is a major factor leading to the occurrence of SA, which has been confirmed by a large number of studies (11, 64, 65). Furthermore, a study on an MR analysis reported causal effects of obesity on risk of SA (66). Many studies reported a strong association between lipids levels and obesity (67, 68). Two reviews pointed out that obesity was an important factor increasing the prevalence of hypertriglyceridemia (67, 68). Therefore, we considered the impact of obesity in the causal effects of triglycerides on the risk of SA. Before the MR analysis, we searched all the traits of SNPs in the primary instrumental variables from PhenoScanner and excluded all SNPs related to obesity. The removed SNPs are shown online in Supplementary Table 1. Through this procedure, we demonstrated that the causal impact of triglycerides on risk of SA was significant without the confounding effects of obesity.

The interaction between lipid profiles should also be considered. MVMR analysis removes interfering factors effectively. By MVMR, we also found significant causal effects of genetically increased triglycerides on risk of SA, but no significant causal relationship was found with HDL, LDL, ApoA-1, and ApoB. The results provided stronger support for the causal relationship we found. In addition, we also considered that SA might be influenced by age, sex, and airway obstruction. The related multivariable Mendelian randomization analysis indicated that the causal effects of triglycerides on SA were not affected by the confounding factors, namely, age, sex, and airway obstruction.

One limitation of our study was that although the sensitivity analysis did not detect horizontal pleiotropy, confounding and pleiotropic factors may still exist (31). For example, we only removed SNPs related to the trait obesity, because we only found a causal relationship between obesity and SA risk in previous studies based on MR analysis (66). However, SA might be associated with many systemic diseases (2–4), and the incorporated SNPs in the lipid profile instruments might still impact SA risk through other ways. Another limitation was that our study was mainly based on a GWAS, whose participants were European, on the UK-Biobank, and different types of studies based on other populations need to be further analyzed.

## Conclusion

The present study demonstrates the independent causal effects of genetically increased triglycerides on risk of sleep apnea without the confounding effects of obesity by two-sample Mendelian randomization and multivariable Mendelian randomization, and suggests that lowering triglyceride concentration contributes to the reduction in risk of sleep apnea.

## Data Availability Statement

The original contributions presented in this study are included in the article/Supplementary Material, further inquiries can be directed to the corresponding authors.

## Author Contributions

HT contributed in software application, resources, and wrote the manuscript. QZ contributed in methodology and data curation. FZ and TW contributed in preliminary investigation. JJ contributed to editing the manuscript and supervision. Y-DT contributed to editing the manuscript. All authors contributed to the article and approved the submitted version.

## Conflict of Interest

The authors declare that the research was conducted in the absence of any commercial or financial relationships that could be construed as a potential conflict of interest.

## Publisher’s Note

All claims expressed in this article are solely those of the authors and do not necessarily represent those of their affiliated organizations, or those of the publisher, the editors and the reviewers. Any product that may be evaluated in this article, or claim that may be made by its manufacturer, is not guaranteed or endorsed by the publisher.

## Footnotes

1
http://www.phenoscanner.medschl.cam.ac.uk/

2
https://gwas.mrcieu.ac.uk/

3
http://www.bristol.ac.uk/integrative-epidemiology/

## Supplementary Material

Supplementary Figure 1 Forest plot. **(A)** Triglyceride, **(B)** high-density lipoprotein, **(C)** low-density lipoprotein, **(D)** apolipoprotein A-1, and **(E)** apolipoprotein B.

Supplementary Figure 2 Funnel plot. **(A)** Triglyceride, **(B)** high-density lipoprotein, **(C)** low-density lipoprotein, **(D)** apolipoprotein A-1, and **(E)** apolipoprotein B.

Supplementary Table 1 Removal of single nucleotide polymorphisms (SNPs) associated with potential confounders.

Supplementary Table 2 Final extracted SNPs.
