# Supplementary figures and images for: The Causal Effects of Lipid Profiles on Sleep Apnea
Source: Front Nutr. 2022 Jun 21;9:910690. doi: 10.3389/fnut.2022.910690 (PMC9253611; doi:10.3389/fnut.2022.910690)

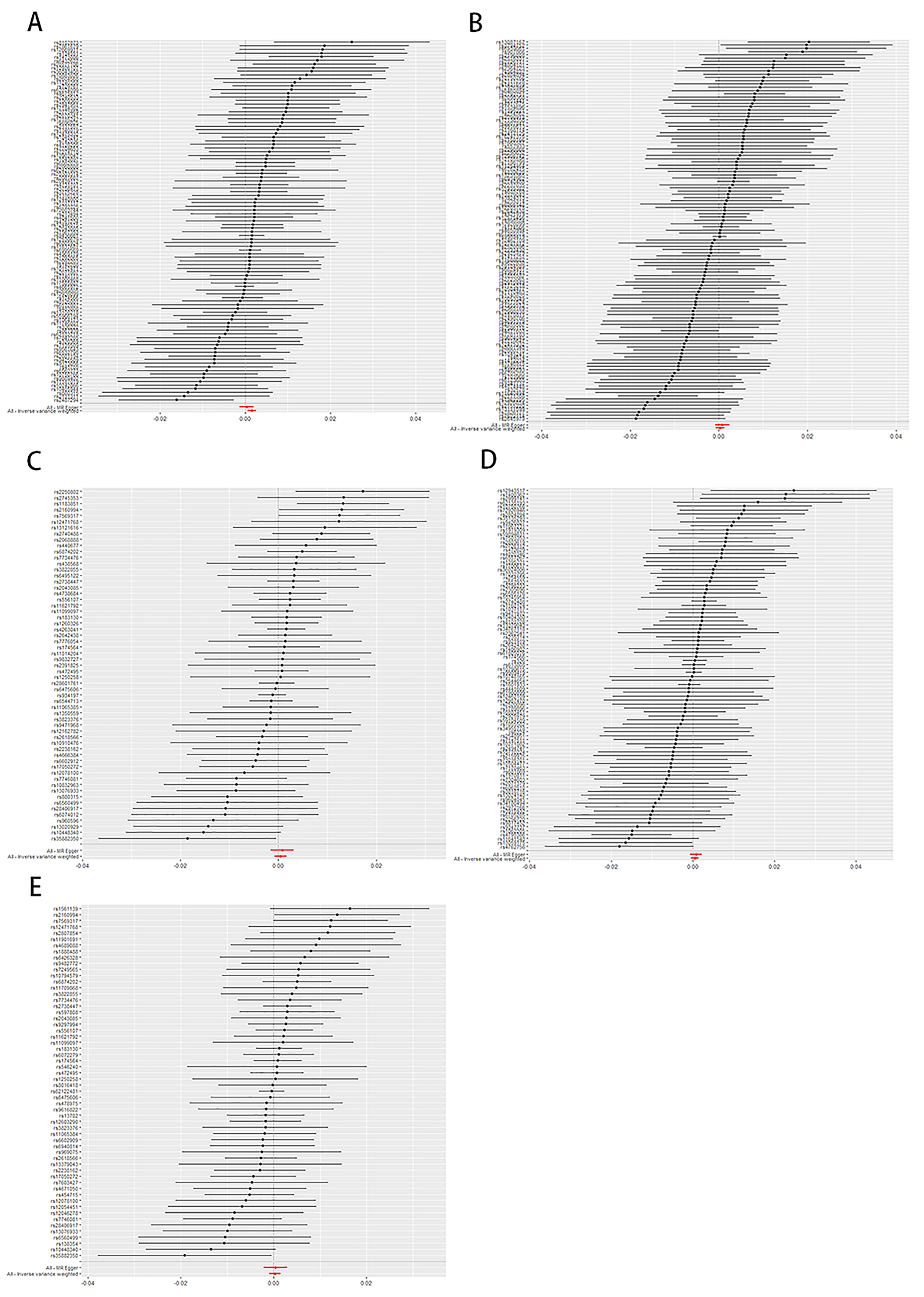

Supplement: Supplementary Figure 1 — Forest plot. (A) Triglyceride, (B) high-density lipoprotein, (C) low-density lipoprotein, (D) apolipoprotein A-1, and (E) apolipoprotein B. [file Image_1.TIF]

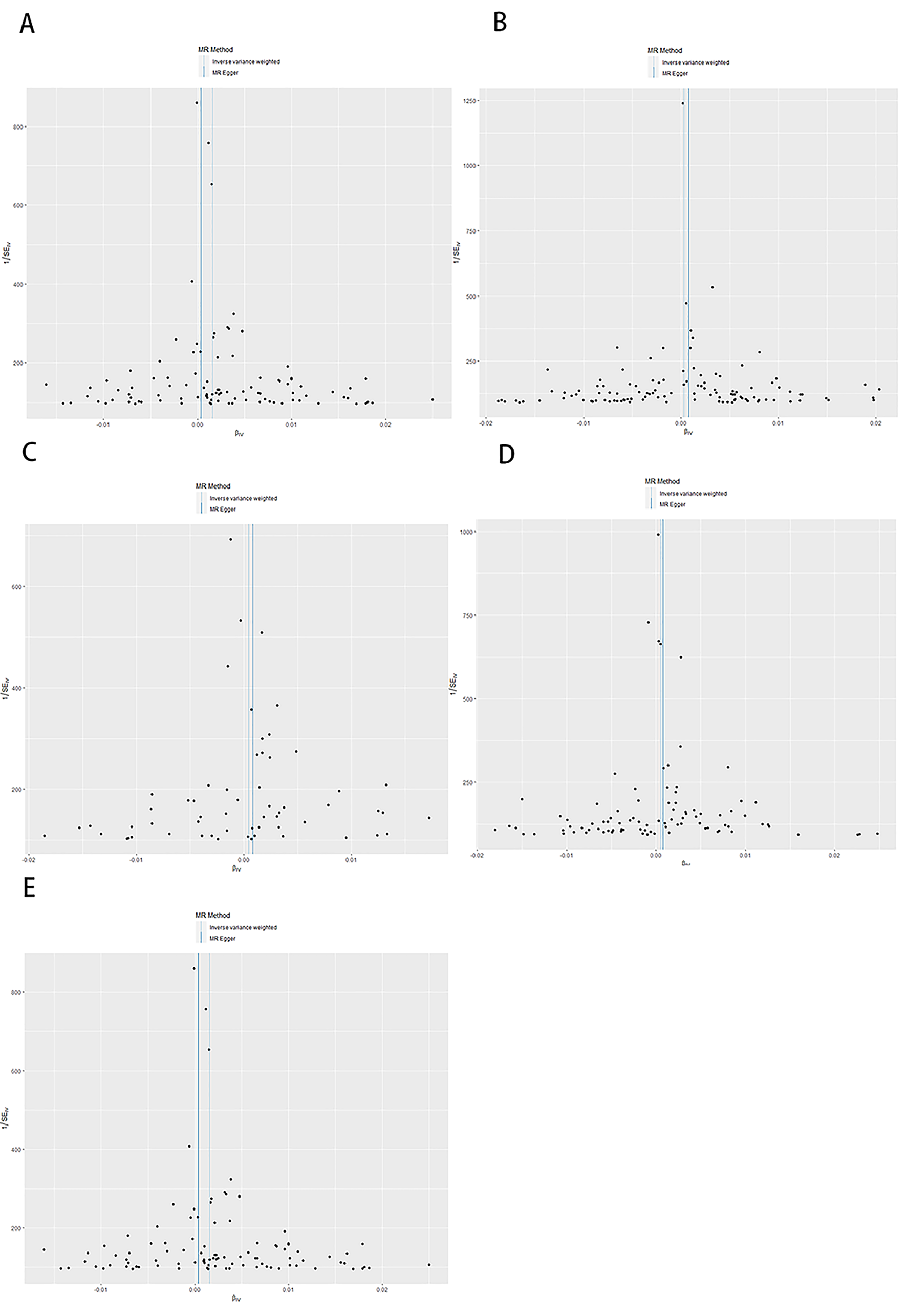

Supplement: Supplementary Figure 2 — Funnel plot. (A) Triglyceride, (B) high-density lipoprotein, (C) low-density lipoprotein, (D) apolipoprotein A-1, and (E) apolipoprotein B. [file Image_2.TIF]
